# Autonomous underwater vehicle fault diagnosis dataset

**DOI:** 10.1016/j.dib.2021.107477

**Published:** 2021-10-14

**Authors:** Daxiong Ji, Xin Yao, Shuo Li, Yuangui Tang, Yu Tian

**Affiliations:** a The Key Laboratory of Ocean Observation-Imaging Testbed of Zhejiang Province, The Institute of Marine Electronic and Intelligent System, Ocean College, Zhejiang University, China; b The Engineering Research Center of Oceanic Sensing Technology and Equipment, Ministry of Education, Zhoushan 316000, China; c Shenyang Institute of Automation, Chinese Academy of Sciences, Shenyang, Liaoning 110016, China

**Keywords:** Fault diagnosis, Autonomous underwater vehicles (AUV), Model-free, State data, Fault type

## Abstract

The dataset contains 1225 data samples for 5 *fault types* (labels). We divided the dataset into the training set and the test set through random stratified sampling. The test set accounted for 20% of the total dataset. Our experimental subject is ‘Haizhe’, which is a small quadrotor AUV developed in the laboratory. For each *fault type*, ‘Haizhe’ was tested several times. For each time, ‘Haizhe’ ran the same program and sailed underwater for 10–20 s to ensure that *state data* was long enough. The *state data* recorded in each test were then used as a data sample, and the corresponding *fault type* was the true label of the data sample. The dataset was used to validate a model-free fault diagnosis method proposed in our paper [1] and the complete dynamic model of ‘Haizhe’ AUV was reported in [2].

## Specifications Table

**Table:** 

Subject	Ocean and Maritime Engineering
Specific subject area	Fault Diagnosis of Autonomous Underwater Vehicles
Type of data	Table
How data were acquired	We used ‘Haizhe’ AUV as the experimental subject. ‘Haizhe’ is installed with 4 brushless motors (SUNNYSKY A2212 KV980 II), 4 propellers (Three-bladed Propeller Outer Diameter: 55mm,Thread Pitch: 80mm), 4 electronic speed control (HOBBYWING Skywalker 20A), 1 depth sensor (MS5803-01 BA), 1 nine-axis inertial measurement unit (GY-MPU9250), and 1 microcontroller unit (STM32F407VET6).
Data format	Raw
Parameters for data collection	We set five common *fault types* for ‘Haizhe’, including normal state, slight damage to the propeller, severe damage to the propeller, failure of the depth sensor, and load increase. In the experiment, ‘Haizhe’ had only one *fault type*at the same time, and there was no multi-failure concurrency. The fault of depth sensor was not the hardware damage but artificially added a bias item when reading the pressure value, which made the calculated depth value deviate from the true value. For example, when the true depth was 0.5m, the depth calculated by the sensor was 0.6m. ‘Haizhe’ sailed underwater for 10-20 seconds each time.
Description of data collection	Firstly, the *fault type* was set and recorded for the ‘Haizhe’. Then, the initializer was executed to check whether each component could work properly or not. After that, ‘Haizhe’ executed the main program and turned on the function of data recording. And then ‘Haizhe’ began sailing underwater. It is worth noting that different behavioral responses would be generated under the influence of different *fault types*, but the main program did not change. After completing the main program, ‘Haizhe’ would stop data recording and automatically rose to the surface. Finally, the file system of ‘Haizhe’ would save the *state data* as a text file (also called a data sample). For each fault type, ‘Haizhe’ was tested several times. The *state data* recorded in each test were then used as a data sample, and the corresponding *fault type* was the true label of the data sample.
Data source location	Institution: The Institute of Marine Electronics and Intelligent Systems, Ocean College, Zhejiang University City/Town/Region: Zhoushan Country: China
Data accessibility	https://doi.org/10.17632/7rp2pmr6mx.1
Related research article	https://doi.org/10.1016/j.oceaneng.2021.108874.

## Value of the Data

• Our paper proposed a diagnosis model for AUV, which could learn the potential pattern between *state data* and *fault type* from the dataset. We hope that more researchers will pay attention to our approach and propose a better diagnosis model. The submitted dataset is recorded in the experiment, which can be used as a standard dataset to verify the performance of the diagnosis model. Although, the submitted dataset is not big enough, we will gradually collect more samples.
• Those who research on fault diagnosis of autonomous underwater vehicle or want to analyze the correlation between *state data* and *fault type* can benefit from this dataset.
• The submitted dataset includes training set and test set. Researchers can train their diagnosis model from training set and use the test set to validate the performance of the trained model. In addition, researchers can also use statistical knowledge or machine learning for data mining on this dataset.

## Data Description

In the submitted dataset, the folder (named “Dataset”) contains two folders (one is named “train”, another is named “test”). “train” means training dataset, while “test” means test dataset. Both folders contain 5 folders: “AddWeight”, “Normal”, “PressureGain constant”, “PropellerDamage bad” and “PropellerDamage slight”. “AddWeight” corresponds to load increase (*fault type*); “Normal” corresponds to normal state; “PressureGain constant” corresponds to failure of the depth sensor; “PropellerDamage bad” corresponds to severe damage to the propeller; “PropellerDamage slight” corresponds to slight damage to the propeller.

Each *fault type* folder contains data samples. And each sample is a.csv file, which records *state data* of ‘Haizhe’ over a certain period of time. The name of *fault type* folder represents the true label of the sample. There are 17 columns in a.csv file. The name and description of each column is listed as below:

• time: The absolute time for ‘Haizhe’ to record data.
• pwm1: Duration (in microseconds) of high level in 100 Hz PWM wave. It is the control signal used to control the Motor 1.
• pwm2: Duration (in microseconds) of high level in 100 Hz PWM wave. It is the control signal used to control the Motor 2.
• pwm3: Duration (in microseconds) of high level in 100 Hz PWM wave. It is the control signal used to control the Motor 3.
• pwm4: Duration (in microseconds) of high level in 100Hz PWM wave. It is the control signal used to control the Motor 4.
• depth: The depth value (in meters) measured by depth sensor.
• press: The pressure value (in Pa) measured by depth sensor.
• voltage: The voltage value (in V) of battery.
• roll: The roll angles (in degrees) measured by nine-axis IMU.
• pitch: The pitch angles (in degrees) measured by nine-axis IMU.
• yaw: The yaw angles (in degrees) measured by nine-axis IMU.
• a_x: The acceleration (in ${\text{m/s}}^{2}$) along the x-axis in the body coordinate frame, measured by nine-axis IMU.
• a_y: The acceleration (in ${\text{m/s}}^{2}$) along the y-axis in the body coordinate frame, measured by nine-axis IMU.
• a_z: The acceleration (in ${\text{m/s}}^{2}$) along the z-axis in the body coordinate frame, measured by nine-axis IMU.
• w_row: The angular velocity (in degrees/s) of rotation around the x-axis in the body coordinate frame, measured by nine-axis IMU.
• w_pitch: The angular velocity (in degrees/s) of rotation around the y-axis in the body coordinate frame, measured by nine-axis IMU.
• w_yaw: The angular velocity (in degrees/s) of rotation around the z-axis in the body coordinate frame, measured by nine-axis IMU.

The models of the vehicle are developed in another work which may be published in the journal of Ocean Engineering. The paper’s title is “Dynamic Modeling of Quadrotor AUV Using a Novel CFD Simulation”.

Note: It is kindly remind that any paper that uses the Data should cite [2] and the above paper if it is published.

## Experimental Design, Materials and Methods

**Fig. 1:**
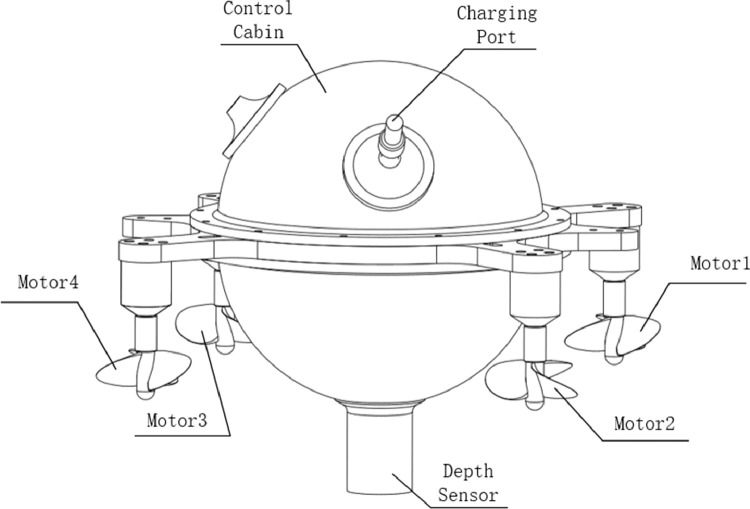
“Haizhe” assembly diagram.

**Fig. 2:**
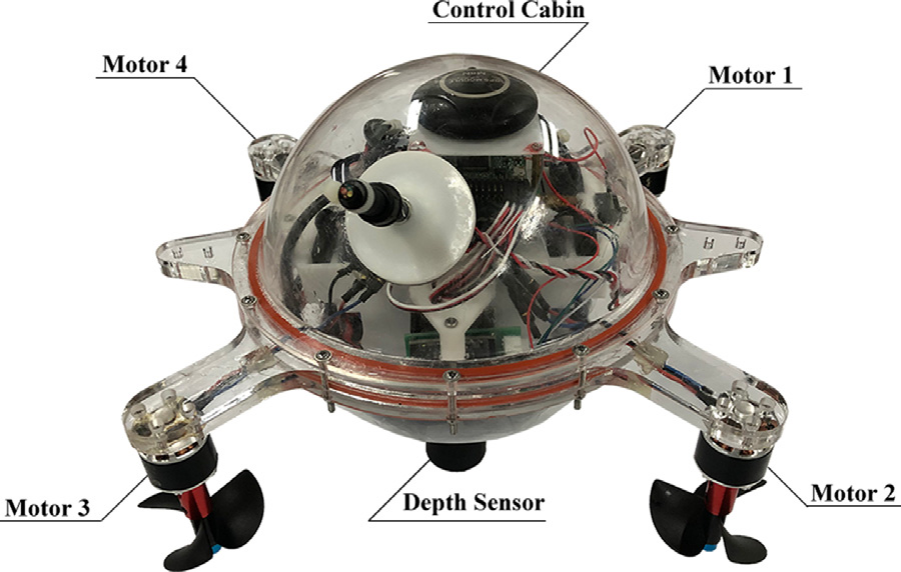
The prototype of ‘Haizhe’.

We used ‘Haizhe’ AUV as the experimental subject. Fig. 1 is the assembly diagram. Fig. 2 is the actual prototype. ‘Haizhe’ is installed with 4 brushless motors (SUNNYSKY A2212 KV980 II), 4 propellers (Three-bladed Propeller Outer Diameter: 55 mm,Thread Pitch: 80mm), 4 electronic speed control (HOBBYWING Skywalker 20A), 1 depth sensor (MS5803-01 BA), 1 nine-axis inertial measurement unit (GY-MPU9250), and 1 microcontroller unit (STM32F407VET6). More detailed information of main components is listed below:

**Table:** 

Main Components	Type

Microcontroller Unit	STM32F407VET6
Brushless DC Motor	SUNNYSKY A2212 KV980 II
Propeller (Counterclockwise)	Three-blade paddle (Outer Diameter: 55 mm, Thread Pitch: 80 mm)
Propeller (Clockwise)	Three-blade paddle (Outer Diameter: 55 mm, Thread Pitch: 80 mm)
Electronic Speed Control	HOBBYWING SkyWalker 20A
Battery	Sony Power Battery VTC5
Depth Sensor	MS5803-01 BA
Nine Axis IMU	GY-MPU9250
2.4G Wireless Module	EBYTE E34 2G4D20D
SD Card	Kingston 16g SD Card

**Fig. 3:**
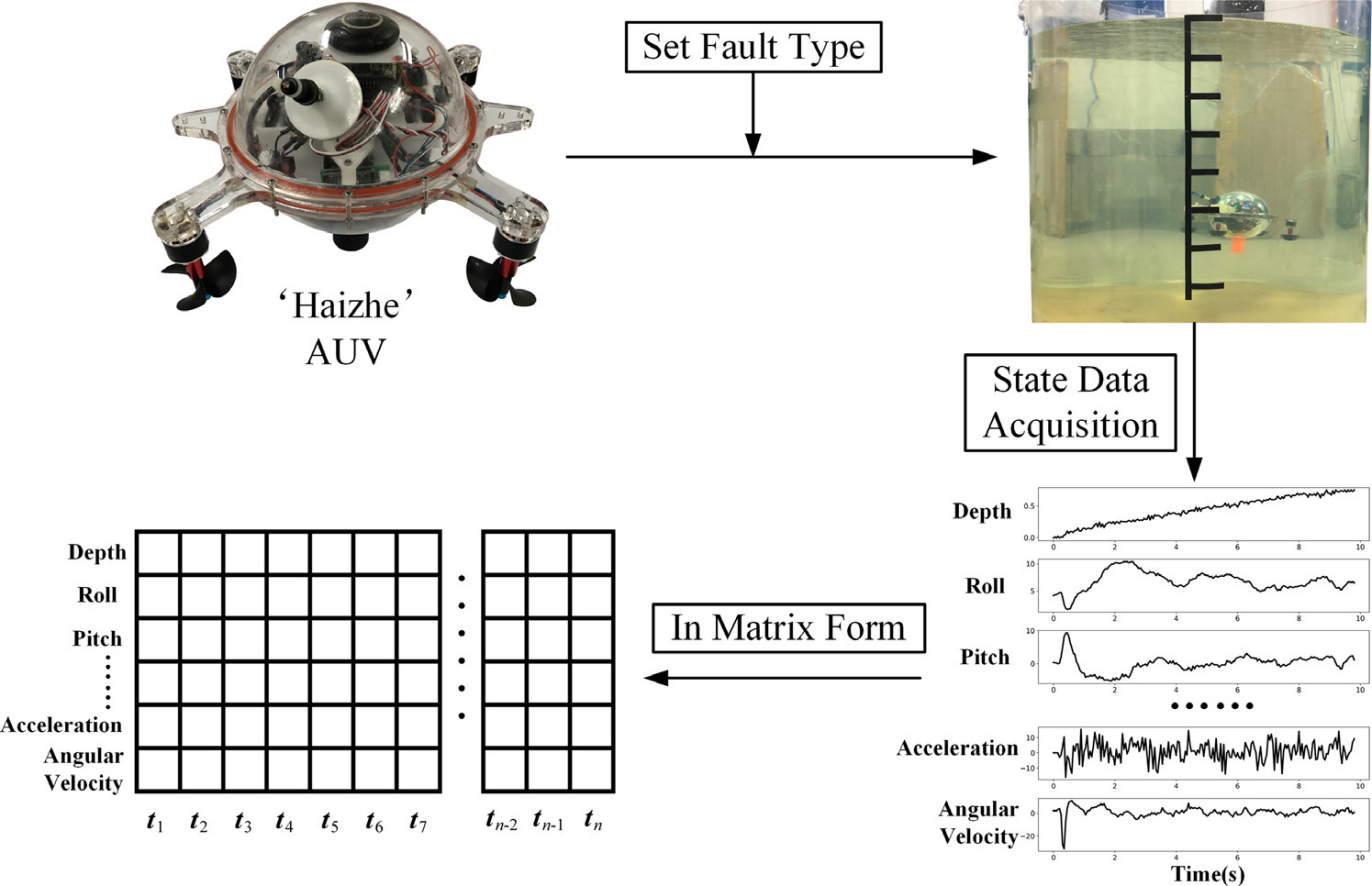
A complete data collection test of ‘Haizhe’.

Figure 3 shows a complete data collection test of the ‘Haizhe’ AUV. Firstly, the *fault type* was set and recorded for the ‘Haizhe’. Then, the initializer was executed to check whether each component could work properly or not. After that, ‘Haizhe’ executed the main program and turned on the function of data recording. And then ‘Haizhe’ began sailing underwater. It is worth noting that different behavioral responses would be generated under the influence of different *fault types*, but the main program did not change. After completing the main program, ‘Haizhe’ would stop data recording and automatically rose to the surface. Finally, the file system of ‘Haizhe’ would save the *state data* as a text file (also called a data sample).

We set five common *fault types* for ‘Haizhe’, including normal state, slight damage to the propeller, severe damage to the propeller, failure of the depth sensor, and load increase. For each *fault type*, ‘Haizhe’ was tested several times. For each time, ‘Haizhe’ ran the same program and sailed underwater for 10–20 s to ensure that *state data* was long enough. The *state data* recorded in each test were then used as a data sample, and the corresponding *fault type* was the true label of the data sample.

In the experiment, ‘Haizhe’ had only one *fault type* at the same time, and there was no multi-failure concurrency. The fault of depth sensor was not the hardware damage but artificially added a bias item when reading the pressure value, which made the calculated depth value deviate from the true value. For example, when the true depth was 0.5 m, the depth calculated by the sensor was 0.6 m.

## Ethics Statement

We declare that the manuscript adheres to Ethics in publishing standards and the submitted dataset is the real data recorded in the experiment, and there is no act of stealing other people’s data or modifying data.

## CRediT authorship contribution statement

**Daxiong Ji:** Conceptualization, Methodology. **Xin Yao:** Software, Data curation, Writing – original draft. **Shuo Li:** Investigation. **Yuangui Tang:** Supervision. **Yu Tian:** Validation.

## Declaration of Competing Interest

We declare that we have no known competing financial interests or personal relationships which have, or could be perceived to have, influenced the work reported in this article.
